# Effect of a community-based diabetes self-management empowerment program on mental health-related quality of life: a causal mediation analysis from a randomized controlled trial

**DOI:** 10.1186/s12913-015-0779-2

**Published:** 2015-03-22

**Authors:** Takehiro Sugiyama, William Neil Steers, Neil S Wenger, Obidiugwu Kenrik Duru, Carol M Mangione

**Affiliations:** Department of Medicine, Division of General Internal Medicine and Health Services Research, David Geffen School of Medicine at UCLA, 3rd Floor, 911 Broxton Ave, Los Angeles, California 90024 USA; Department of Clinical Study and Informatics, Center for Clinical Sciences, National Center for Global Health and Medicine, 1-21-1 Toyama Shinjuku-ku, Tokyo, 162-8655 Japan; Department of Public Health/Health Policy, Graduate School of Medicine, The University of Tokyo, 7-3-1, Hongo, Tokyo, Bunkyo-ku 113-0033 Japan; Department of Health Policy and Management, UCLA Fielding School of Public Health, 640 Charles E Young Dr S, Los Angeles, California 90024 USA

**Keywords:** Randomized controlled trial, Diabetes mellitus, Self-management, Patient education, Mental health

## Abstract

**Background:**

There is a paucity of evidence supporting the effectiveness of diabetes self-management education (DSME) in improving mental health-related quality of life (HRQoL) for African American and Latinos. Also, among studies supporting the favorable effects of DSME on mental HRQoL, the direct effect of DSME that is independent of improved glycemic control has never been investigated. The objectives of this study were to investigate the effect of community-based DSME intervention targeting empowerment on mental HRQoL and to determine whether the effect is direct or mediated by glycemic control.

**Methods:**

We conducted secondary analyses of data from the Diabetes Self-Care Study, a randomized controlled trial of a community-based DSME intervention. Study participants (n = 516) were African Americans and Latinos 55 years or older with poorly controlled diabetes (HbA1c ≥ 8.0%) recruited from senior centers and churches in Los Angeles. The intervention group received six weekly small-group self-care sessions based on the empowerment model. The control group received six lectures on unrelated geriatrics topics. The primary outcome variable in this secondary analysis was the change in Mental Component Summary score (MCS-12) from the SF-12 Health Survey between baseline and six-month follow-up. We used the change in HbA1c during the study period as the main mediator of interest in our causal mediation analysis. Additionally, possible mediations via social support and perceived empowerment attributable to the program were examined.

**Results:**

MCS-12 increased by 1.4 points on average in the intervention group and decreased by 0.2 points in the control group (difference-in-change: 1.6 points, 95% CI: 0.1 to 3.2). In the causal mediation analysis, the intervention had a direct effect on MCS-12 improvement (1.7 points, 95% CI: 0.2 to 3.2) with no indirect effects mediated via HbA1c change (−0.1 points, 95% CI: −0.4 to 0.1), social support (0.1 points), and perception of empowerment (0.1 points).

**Conclusions:**

This Diabetes Self-Care Study empowerment intervention had a modest positive impact on mental HRQoL not mediated by the improvement in glycemic control, as well as social support and perception of empowerment. This favorable effect on mental HRQoL may be a separate clinical advantage of this DSME intervention.

**Trial Registration:**

ClinicalTrial.gov NCT00263835.

**Electronic supplementary material:**

The online version of this article (doi:10.1186/s12913-015-0779-2) contains supplementary material, which is available to authorized users.

## Background

Diabetes is an important public health concern not only because of the direct morbidity and mortality that it causes, but also because it contributes to many other health problems including micro- and macro-vascular diseases. Diabetes disproportionately affects African American and Latino populations, probably through both socioeconomic and genetic factors [1]. Diabetes is also linked to poorer mental functioning, as evidenced by its association with lower mental health-related quality of life (HRQoL) in population-based research [2]. The presence of diabetes doubled the odds of comorbid depression in a published meta-analysis [3]. The causal relationship between diabetes and impaired mental health is thought to be bidirectional, with each condition exacerbating the other [4]. Therefore, when healthcare providers see a patient with diabetes, they should screen for depression and other diabetes-related psychosocial problems and, if present, treat them because these problems may interfere with maintaining control of diabetes [5]. There is also an increasing interest in the relationship between diabetes treatment options and psychological outcomes [6].

**Figure 1:**
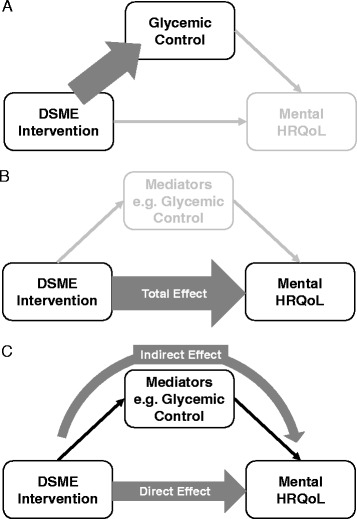
**Comparison of conceptual models. A**. Original research question of Diabetes Self-Care Study – effect of the DSME intervention on glycemic control. **B**. First research question of our research – total effect of the DSME intervention on mental health-related quality of life (mental HRQoL). **C**. Second and third research questions of our research – direct effect of the DSME intervention on mental HRQoL separate from indirect effect via glycemic control (second research question) or other mediators (third research question). DSME = Diabetes self-management education. Total Effect = Direct Effect + Indirect Effect.

Self-management is an integral part of controlling diabetes. For example, optimal glucose control in most cases requires patients to maintain healthy eating and appropriate exercise. Diabetes self-management education (DSME) has been described as “a collaborative process through which people with or at risk for diabetes gain the knowledge and skills needed to modify behavior and successfully self-manage the disease and its related conditions [7]”. There is considerable evidence that DSME improves glycemic control, albeit modestly (Figure 1A) [8,9]. In addition, several researchers have hypothesized that DSME improves psychological aspects through a positive attitude toward health and increased diabetes self-efficacy (Figure 1B). For example, Kirk et al. reported that exercise consultation compared to standard care improved the mental health subscale of SF-36 5 weeks after the intervention in a small randomized controlled trial (RCT) among patients with Type 2 diabetes (T2DM) [10] However, other studies of DSME showed either no effect or worsened mental HRQoL [11-13]. A few systematic reviews and meta-analyses attempted to assess the effect of DSME on generic and diabetes-specific quality of life measures [9,14]; however, they could draw no conclusion related to quality of life due to heterogeneity of included studies or a paucity of studies measuring quality-of-life. In summary, both the type and extent of the effect of DSME on mental quality of life among patients with diabetes remain unclear. Evidence within African American and Latino populations is particularly scarce.

Furthermore, if DSME favorably affects mental health, it is crucial to understand whether this effect is derived directly from DSME or indirectly through improvement of other factors (Figure 1C) in order to elucidate the intervening mechanism of DSME. We are especially interested in the possible mediation by glycemic control; even though RCTs showed improvements in both glycemic control and quality of life, additional analysis is needed to investigate whether the improved quality of life is associated with glycemic control. Very few studies have investigated whether the effect of other types of diabetes interventions on psychological outcomes are mediated by glycemic control [15,16], but these employed basic analytic approaches such as ordinary linear regressions (Baron and Kenny’s mediation analysis [17]) and bivariate correlations. Methods such as structural equation modeling [18] and its derivative causal mediation analysis [19], and Bayesian network analysis [20] are useful for quantitatively estimating a direct effect of the intervention on the psychosocial outcome not mediated by the proximate clinical outcomes.

In these analyses using data from the Diabetes Self-Care Study [21], we investigated (i) the effect of a community-based group DSME program on mental HRQoL. We also investigated (ii) whether the DSME intervention had a direct effect on mental HRQoL that was separate and distinct from an indirect effect mediated by improved glycemic control, which was the main research question of our study. We additionally tested whether (iii) other mediators including social support and perceived empowerment resulting from DSME might explain the link between the DSME intervention and improved mental HRQoL. We tested perceived empowerment as a mediator because the DSME intervention was grounded in empowerment theory [22], and empowerment theory posits that DSME should lead to improved outcomes because it increases levels of empowerment to control one’s illness. We also investigated (iv) whether the level of social support moderated the effect of the DSME intervention on mental HRQoL.

Based on our review of the literature and our clinical experience, we hypothesized the following: (i) the DSME intervention of our study is likely to improve mental HRQoL in this population overall, (ii) the beneficial effect of the DSME intervention on mental HRQoL is likely to be direct and not mediated by glycemic control, (iii) the beneficial effect of the DSME intervention on mental HRQoL is likely to be specifically mediated by increases in improvement in social support and participants’ perceived empowerment resulting from the DSME intervention, and (iv) the effects of the DSME intervention on MCS-12 will be strongest among participants with high social support because they are better able to implement what they learned in DSME.

## Methods

### Design, settings and participants

This study analyzed data from the Diabetes Self-Care Study, a RCT that investigated the effectiveness of community-based DSME in older African Americans and Latinos with diabetes (NCT00263835, ClinicalTrial.gov). The original research question of the study was whether a community-based DSME leads to better glycemic control among participants.

Participants in the Diabetes Self-Care Study were recruited from senior centers, churches, community clinics, and Los Angeles County Community and Senior Service Centers between November 2003 and October 2005. Inclusion criteria for this study were as follows: HbA1c of 8% or greater, African American race or Latino ethnicity, community-dwelling or living in close proximity to study site, proficiency in English and/or Spanish, and adequate cognition to participate in the study. Patients with at least moderate disabilities, those who were pregnant, and those already enrolled in diabetes classes were excluded. We previously showed that this DSME program resulted in a statistically significant improvement HbA1c when compared to the control arm of the study ([21], also shown in the Results section). We included all the samples analyzed in the study about glycemic control, but a part of the samples were excluded due to missing data for the primary outcome (see the CONSORT flowchart in the Website Appendix for a summary of how the analytic sample was obtained).

The Diabetes Self-Care Study including this secondary analysis was approved by the UCLA IRB (#G00-03-079-11). Informed consent was obtained from all participants.

### Study process and interventions

All study participants were given glucose meters and testing strips, and received a 2-hour training on self-monitoring of blood glucose by a certified diabetes educator. Participants then were randomly assigned to either the intervention or the control group on an individual basis using sealed envelops with cards marked either “Study” or “Control”. Participants were made aware of their allocation status and were provided with an explanation of the details of their arm of the study. Participants in the intervention group received six weekly two-hour group self-care sessions consisting of 8 to 10 persons per group, conducted in English or Spanish, and facilitated by health educators. Health educators completed a one-year training program and received 8 hours of curricula delivered by the study team about diabetes and its clinical presentations and complications. Additionally, they received 12 hours of training and implementation of the empowerment sessions. Throughout the study, sessions were audiotaped and were reviewed for fidelity. In the group session, participants identified self-management challenges and discussed why each activity was challenging and how to solve it. After the discussion, each participant identified a “personal experiment” in which she/he would try to overcome a particular barrier, and at the next session, she/he shared the result of the “experiment” and modified it if they were not initially successful. The intervention group also was offered a video to jump-start the discussion, if needed, and each participant was given a pictorial workbook written at the sixth grade level that covered a wide array of self-care topics and provided templates for recording personal experiments and their outcomes. Each participant also had a one-on-one session with the health educator to review his or her baseline and follow-up laboratory and biometric data during one of the group sessions. Additionally, with the participants’ consent, a report of all laboratory and biometric findings were shared with their regular doctors. The control group received six weekly two-hour lectures on unrelated geriatric topics to balance contact with the research staff. Additionally, the control group had one-on-one sessions to review their laboratory and biometric data and had the opportunity to have the study team share their results with their physician.

### Measurements

Baseline survey and laboratory and physical examinations were conducted before randomly assigning participants to groups. Follow-up survey and examinations were performed approximately six months after the last session or lecture of each group. Our primary outcome was the change in the Mental Component Summary Score (MCS-12) from the SF-12 Health Survey from baseline to six-month follow-up [23]. MCS-12 and the Physical Component Summary Score (PCS-12) are measures of mental and physical HRQoL, which are calculated from SF-12 items and are standardized with means of 50 and SDs of 10 in the general population. Participants completed the SF-12 at baseline and at six-month follow-up, and changes in MCS-12 scores over time were compared between the groups. Potential mediators of change in MCS-12 included social support and perceived empowerment resulting from the DSME program. Social support was measured with the six-item domain of the Diabetes Care Profile [24] about diabetes-related support received from family or friends; the summary score ranges from 1 (least support) to 5 (most support). The social support measurement was measured at baseline and six-month follow-up. At baseline, questions asking about participants’ background characteristics were included. At six-month follow-up, participants completed 5 separate five-point Likert items asking about the level of empowerment resulting from the DSME program as follows:

- This program has helped me feel better.
- Since starting this program, I feel more confident in my ability to manage my diabetes.
- Since starting this program, I feel more confident in my ability to communicate with my doctor about my diabetes.
- Since starting this program, I have improved the way I manage my diabetes.
- Since starting this program, I have become better at solving problems related to managing my diabetes.

These items showed high internal consistency reliability (alpha = 0.92); we used the average of these scores, which ranged from 1 (strongly disagree = least empowered) to 5 (strongly agree = most empowered). Spanish language version of the SF-12 Health Survey were used for Spanish-speaking participants. For the measures and questionnaires where Spanish translation did not exist, we conducted forward-backward translation to verify the validity.

HbA1c, the primary outcome of the original RCT, was measured at baseline and six-month follow-up. All participants had their blood drawn by the study team. HbA1c was measured in the UCLA Clinical Laboratory.

All measurements were conducted for both the intervention and control groups in the same way. Each participant, whether in the treatment or control group, received a $10 gift card for each assessment whereas no incentive was awarded for attendance at a session.

### Statistical analysis

First, we generated descriptive statistics comparing the pre-intervention characteristics for intervention versus control groups. These characteristics included demographic, biometric, and psychological measures. Then, we compared the changes from baseline to six-month follow up of HbA1c, PCS-12, and MCS-12 between the two groups. This unadjusted comparison of change in MCS-12 addressed our first research question about the overall effect of the DSME intervention on mental HRQoL. We also compared changes in social support, and we compared the groups on perceived empowerment attributable to the program at six-months after completion of the intervention.

To address our second research question, we used a statistical method called causal mediation analysis to estimate the direct effect of the DSME intervention on mental HRQoL versus the indirect effect on mental HRQoL that is mediated by glycemic control. Causal mediation analysis [19,25], a quantitative application of Baron and Kenny’s mediation analysis [17] and a derivative of structural equation modeling [18], enabled us to decompose a total effect into its (natural) direct effect and indirect effect components along with bootstrapped CIs of the effects.

To address our third research question, we performed additional causal mediation analyses to explore whether the effect of the DSME intervention is mediated by improvements in social support and/or perceived empowerment attributable to the program. We conducted two analyses; we included one of the change in social support and the perceived empowerment at six-months as a mediator in each model.

Because causal mediation analysis requires the assumption that there is no confounding by other unmeasured variables that are associated with both the mediator (e.g., glycemic control) and the outcome of interest (e.g., mental HRQoL), we conducted sensitivity analyses to verify the robustness of our mediation analyses in the presence of possible unmeasured confounders. These analyses involved adjusting the effect size estimates and 95% confidence limits for varying degrees of confounding by unmeasured variables (a possible example: tragic life events) [19].

To assess whether social support moderates the effect of the intervention on mental HRQoL, we performed a regression analysis that included the interaction term between the intervention variable and the baseline score for social support. For this analysis, we dichotomized social support into high/low categories with values below 3 classified as low, and 3 or greater classified as high.

Additionally, we performed two post-hoc analyses: comparisons of MCS-12 change 1) among groups restricted to participants whose HbA1c did not improve during the study period and 2) among Latino participants.

Causal mediation analyses were performed using the “mediation” package of software R 2.14.0. The other analyses were conducted using Stata Version 12.

## Results

Among 516 participants, 258 were allocated to the intervention group while the other 258 were allocated to the control group. The average age of each group was 63 years old. Approximately 70% were female and 60% were Latinos. No statistically significant differences in patient demographic characteristics or laboratory measurements were observed between the groups, whereas years with diabetes were marginally longer in the control group on average. In particular, there were no differences in baseline HbA1c between the groups (p = 0.93). Mean MCS-12 at baseline was approximately 45 points in both groups, whereas mean PCS-12 was higher in the intervention group than in the control group (p = 0.027). Participants in both groups had very similar levels of social support (Table 1). Among those who were randomized, 203 in the intervention group and 196 in the control group had complete data on MCS-12 and were included in the test of the total effect of the DSME intervention (see Additional file 1).

**Table 1:** **Baseline characteristics and measurements of participants by group**

	**Intervention Group**		**Control**		**P-value**
			**Group**		
	N*		N*		
Patient characteristics					
Age (years)	258	63.7 ± 6.3	258	63.3 ± 6.8	0.42
Female	258	177 (68.6)	258	189 (73.3)	0.25
Latino†	258	164 (63.6)	258	152 (58.9)	0.28
Annual income below $20,000	223	180 (80.7)	226	170 (75.2)	0.16
High school or higher education	256	99 (38.7)	258	95 (36.8)	0.67
No DM education in previous 12 months	255	181 (71.0)	255	175 (68.6)	0.56
Years with DM (years)	202	12.2 ± 9.1	206	14.0 ± 11.0	0.074
Laboratory data					
HbA1c (%)	257	9.7 ± 1.6	257	9.7 ± 1.7	0.93
SF-12 Scores					
PCS-12 (points)‡	245	42.3 ± 6.6	244	40.9 ± 7.1	0.027
MCS-12 (points)‡	245	45.0 ± 6.5	244	44.9 ± 6.7	0.94
Social support					
Social support score from Diabetes Care Profile (points) §	254	2.9 ± 1.6	256	3.0 ± 1.5	0.44

Data shown as n (%) or mean ± SD. P-values were calculated by chi-square tests or t-tests.

*Sample size varies by item because of missing responses.

†The rest of the participants were African Americans as per inclusion criteria.

‡Mean and SD in general population are 50 and 10, respectively.

§The score ranges from 1 (least support received) to 5 (most support received).

DM = diabetes mellitus, HbA1c = hemoglobin A1c, SF-12: 12-item short form, PCS-12: physical component summary score of SF-12, MCS-12: mental component summary score of SF-12.

As previously reported and described above, the decrease or improvement of HbA1c in the intervention group was greater than that in the control group (difference in change: −0.4%, 95% CI: −0.8% to −0.1%, p = 0.02, Table 2). The social support score did not change during the study period, and there was no significant difference in change in social support between the groups. Participants’ perception of empowerment from the program in the intervention group was slightly, yet significantly, higher than in the control group. MCS-12 improved 1.4 points in the intervention group while it decreased 0.2 points in the control group on average, and the difference in change was statistically significant (1.6 points, 95% CI: 0.1 to 3.2, Table 2). This difference in change is our estimate of the total effect of the DSME intervention on MCS-12.

**Table 2:** **Six-month changes of measurements and participants’ perception of empowerment attributable to the program at six-month follow-up compared between groups**

	**6-month change score**				**Difference of two groups (I – C)**
	**Intervention Group**		**Control Group**		
	N*		N*		
Laboratory data					
HbA1c (% (mmol/l)	224	−1.0 (−1.2 to −0.7)	217	−0.5 (−0.8 to −0.3)	−0.4 (−0.8 to −0.1)
		(−10.5 (−13.2 to −8.0))		(−5.9 (−8.7 to −3.2))	(−4.6 (−8.4 to −0.9))
Social support					
Social support score from Diabetes Care Profile (points)	222	0.2 (−0.0 to 0.4)	217	0.1 (−0.1 to 0.3)	0.1 (−0.2 to 0.4)
SF-12 Scores					
PCS-12 (points)	203	0.6 (−0.4 to 1.6)	196	1.6 (0.6 to 2.6)	−1.0 (−2.4 to 0.4)
MCS-12 (points)	203	1.4 (0.3 to 2.5)	196	−0.2 (−1.3 to 0.9)	1.6 (0.1 to 3.2)
Program evaluation (Post-intervention only)					
Participants’ perception of empowerment attributable to the program at follow-up (points) †	226	4.4 (4.4 to 4.5)	216	4.3 (4.2 to 4.4)	0.1 (0.0 to 0.2)

Data shown as mean (95% CI).

*Sample size varies by item because of missing responses.

†The score ranges from 0 (least empowered) to 5 (most empowered).

I – C: intervention group minus control group, HbA1c = hemoglobin A1c, SF-12: 12-item short form, PCS-12: physical component summary score of SF-12, MCS-12: mental component summary score of SF-12.

Table 3 shows the results of the causal mediation analyses. In the first model testing HbA1c as a mediator, we found evidence of a direct effect of the DSME intervention and no evidence of an indirect effect mediated by HbA1c. We proceeded to test the other possible mediators and we found no evidence that social support or perceived empowerment mediated the effect of the DSME intervention on mental HRQoL. In each case, the direct effect explained essentially all of the total effect of the DSME intervention, while the evidence for an indirect effect was negligible.

**Table 3:** **Causal mediation analyses – decomposition of total effect of DSME intervention on MCS-12 change into direct and indirect effects**

**Possible Mediator**	**Direct Effect**	**Indirect Effect**	Total Effect*
Mediator of main interest			
HbA1c (change score)	1.7 (0.2 to 3.2)	−0.1 (−0.4 to 0.1)	1.6 (0.1 to 3.1)
Other possible mediators			
Social support score from Diabetes Care Profile (change score)	1.6 (0.1 to 3.2)	0.1 (−0.1 to 0.3)	1.7 (0.2 to 3.3)
Participants’ perception of empowerment resulting from program (score obtained at follow-up period)	1.5 (0.0 to 3.2)	0.1 (−0.1 to 0.4)	1.7 (0.2 to 3.3)

*Total effect and 95% CI in each analysis were slightly different because each analysis has different missing samples. Total effect does not always appear to be the sum of direct effect and indirect effect due to rounding.

DSME: diabetes self-management education, MCS-12: mental component summary score of SF-12, HbA1c: hemoglobin A1c.

The sensitivity analyses testing the impact of violations of the no confounding by unmeasured variables assumption showed that violations would need to be severe before our inferences about indirect effects would change, thus confirming the robustness of these analyses to violations of the assumptions of no confounding.

In the analysis investigating whether social support modifies the effect of the DSME intervention on mental HRQoL, the intervention was associated with a 1.2-point larger difference in change of MCS-12 among participants with higher social support at baseline, whereas the intervention was associated with a 2.2-point larger difference in change of MCS-12 among participants with lower social support at baseline. The effect modification was not significant (−1.0, 95% CI: −4.2 to 2.2).

In a post hoc analysis restricted to participants without HbA1c improvement during the study period, the intervention was associated with a non-significant1.9-point larger difference in change of MCS-12 (95% CI: −1.1 to 4.9). The other restricted, post hoc analysis showed that the total effect of the DSME intervention on mental HRQoL among Latinos was statistically significant at 2.3 points (95% CI: 0.3 to 4.4).

## Discussion

Previous studies of DSME have shown positive mild effects on glycemic control, but the effects of DSME on mental health outcomes have been inconclusive. The present study demonstrates that our community-based DSME improves mental HRQoL at six-month follow-up. This effect size (≈0.2 SD) of MCS-12 difference-in-change attributable to the DSME intervention is comparable to the minimal important difference described by Hays et al. [26] and is similar to the difference in MCS-12 between people with and without diabetes reported in a population-level study [2]. These comparisons suggest that the effect size of the DSME intervention on mental HRQoL was small but clinically meaningful, and the provision of the DSME intervention might offset reductions in mental HRQoL that are linked to having diabetes.

This result should be interpreted in the context of past studies, some of which showed a measurable effect of DSME on mental HRQoL or other psychological outcomes [10,27-29], while others did not [11-13]. These differences may relate to heterogeneity with regard to source population and intervention strategy. Further research is needed to clarify which elements of DSME are effective for which populations. Of particular interest is comparison of our data to a study by Tang et al. [13] that conducted an empowerment model-based intervention with participants of similar race/ethnicity and age that did not show an effect of DSME on diabetes-specific QoL. They attributed this negative result partly to a floor effect on their QoL measure—diabetes distress. It might also be attributable to the fact that their QoL measure was different from ours (MCS-12 vs. Diabetes Distress Scale (DDS)) or due to chance. An uncontrolled pre- post design pilot study preceding their controlled evaluation reported a much larger improvement in DDS [30]. Further research is needed to determine the effect of empowerment model-based DSME on this population.

We found that improvement in mental HRQoL achieved by the DSME intervention was not mediated by glycemic control. This means that a favorable effect on mental HRQoL might not be expected by interventions that simply improve glycemic control, as might be achieved using pharmacologic interventions. We believe that this is the first study to show the direct effect of DSME on mental HRQoL that is not mediated by improved glycemic control.

It is interesting to consider our finding along with RCTs about other types of diabetes treatment. Some RCTs of pharmacological interventions showed improvements in both glycemic control and psychological outcomes. Most studies, however, did not test the correlation between glycemic and psychological improvements; in these cases, we are not sure whether glycemic control led to psychological improvements. A RCT about amylin analog pramlintide showed the association between glycemic control and reduced psychological distress, and the authors concluded that “the difference between treatment arms in reduction of diabetes-related distress can be attributed to the differential impact of treatment on glucose control” [16]. Their conclusion contrasts with the result of the present study; in our study, the effect of DSME on mental HRQoL was not mediated by improved glycemic control. Based on this comparison, we suppose that DSME may be a complex intervention that has multiple benefits; DSME may improve mental HRQoL regardless of the degree of glycemic control. Clinicians treating patients with chronic conditions should consider a variety of therapeutic strategies that will address not only the proximate clinical outcome, but also psychosocial outcomes such as mental health and self-efficacy.

Additional analyses were unable to tease out the precise mechanism by which the DSME intervention achieved the improvement in mental HRQoL. We found that the effect of the DSME intervention was not exerted through any of several possible mediators, including change in social support and perceived empowerment resulting from the program. These results did not support our hypotheses about likely mediators. Further research is needed to clarify the mechanisms though which the DSME intervention affects mental HRQoL. A possible mediator of this effect that we did not measure in this study was the perceived level of social support from the DSME team of health educators, assistants and other participants. The intervention group received group sessions and interaction with health educators instead of lectures received by the control group, which may have resulted in higher levels of social support among participants and with health educators. The Diabetes Care Profile that asks about social support from family or friends would not have captured support from the study team or other participants.

We also hypothesized that participants with higher social support would enjoy greater benefit of DSME on mental HRQoL than those with lower social support, but we did not find evidence that social support moderates the effect of the DSME intervention on mental HRQoL.

This work has several limitations. First, as described above, causal mediation analysis is susceptible to confounding by common causes of a mediator and an outcome, but the sensitivity analyses confirmed the robustness of our results. Second, we could not rule out the possibility of reverse causality with mental HRQoL as a predictor rather than as an outcome of glycemic control because these data elements were measured in the same time periods (differences between baseline and six-months follow-up). However, our main results about the direct effect would be unchanged even if the relationship between glycemic control and mental HRQoL is bi-directional. Third, approximately 20% of participants in each group dropped out from the study. We conducted an analysis of attrition in order to determine if there was evidence of differential attrition and if those who terminated their participation differed from those who completed the study on any key demographic/clinical variables listed in Table 1; the results of our analysis showed no evidence of differential attrition, or evidence that completers differed from non-completers on any key variables. Fourth, the follow-up period was a relatively brief six months; the long-term effect of DSME on mental HRQoL remains to be fully elucidated. Fifth, this effect may not be generalizable to other racial/ethnic and age groups because all participants were older African Americans and Latinos. However, this study provides data in an area with a paucity of studies addressing the effectiveness of behavioral diabetes interventions on the emotional well-being in Latinos [31]. Notably, the post hoc analysis showed that the DSME intervention improved mental HRQoL even when restricting our sample to Latino participants only. Sixth, as described above, DSME interventions are so heterogeneous that this specific community-based intervention grounded in an empowerment model may not be generalizable to other DSME programs. Seventh, we did not focus on the cost of the intervention because the cost-effectiveness of the intervention was beyond the scope of this study, although we saved costs by introducing health educators without professional license. Finally, the MCS-12 score measures psychological distress, fatigue, social function and role function due to emotional problems, but does not measure depressive symptoms directly. Although several studies have shown that mental HRQoL is associated with measures of depression [32,33], our findings cannot be interpreted as improving depression.

## Conclusions

In conclusion, the Diabetes Self-Care Study empowerment intervention had a small positive impact on mental HRQoL that was not mediated by improved glycemic control among older African Americans and Latinos. This favorable effect of the DSME intervention on mental HRQoL is an important clinical outcome to be considered when treating persons with diabetes.

## Additional file

Additional file 1:
**The CONSORT flowchart of this study.**
