# Supplementary figures and images for: Effect of a community-based diabetes self-management empowerment program on mental health-related quality of life: a causal mediation analysis from a randomized controlled trial
Source: BMC Health Serv Res. 2015 Mar 22;15:115. doi: 10.1186/s12913-015-0779-2 (PMC4375843; doi:10.1186/s12913-015-0779-2)

## Additional file. The CONSORT flowchart of this study.

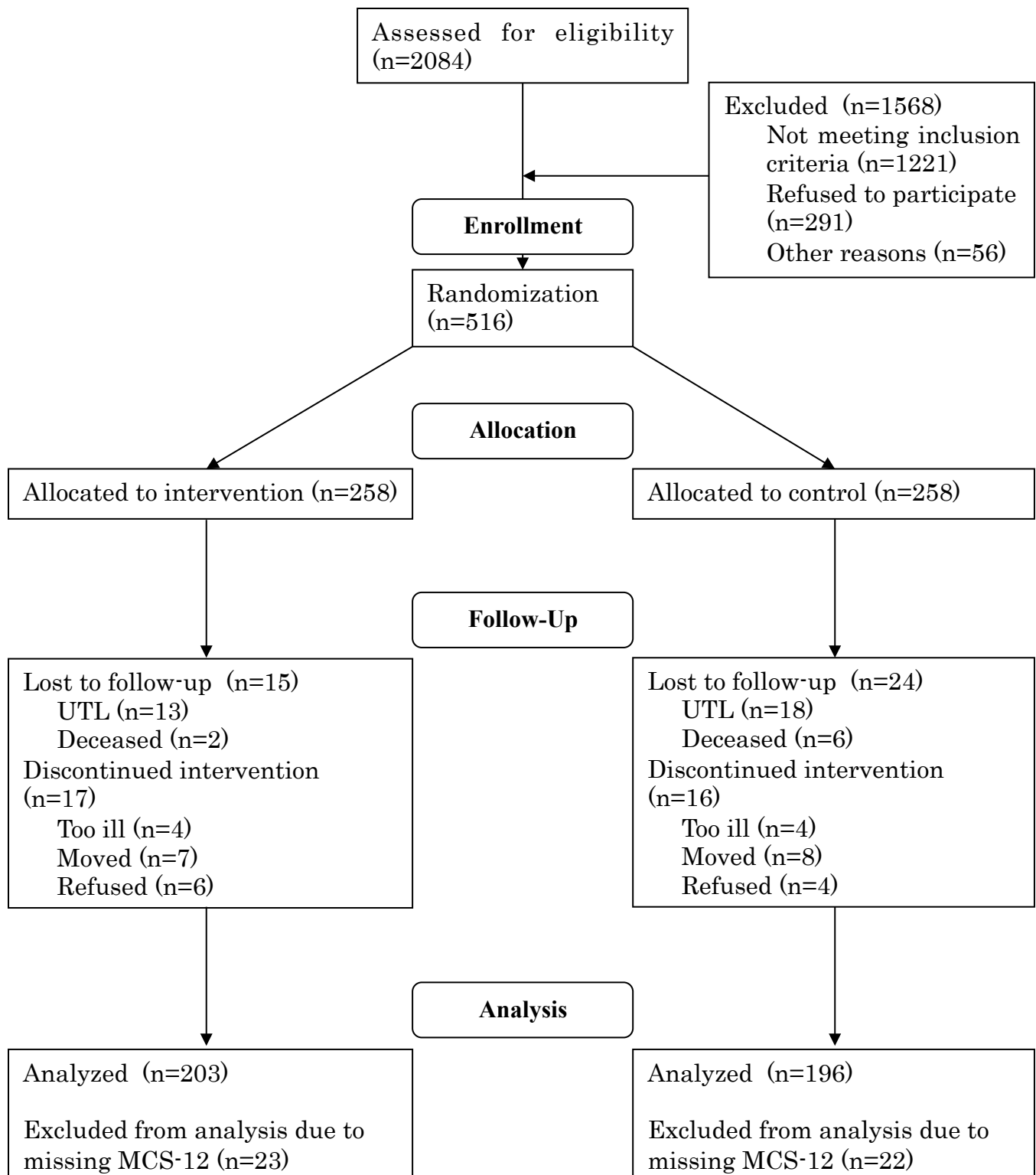

Supplement: Additional file 1: — The CONSORT flowchart of this study. [file 12913_2015_779_MOESM1_ESM.pdf]
